# ARTHROSCOPY LEARNING IN VIRTUAL REALITY: SYSTEMATIC REVIEW AND METANALYSIS

**DOI:** 10.1590/1413-785220253303e287823

**Published:** 2025-08-18

**Authors:** André Luis Lugnani de Andrade, Fernanda Yuri Yuamoto, Claudia Marques Santa Rosa Malcher, Simone Appenzeller, Thiago Alves Garcia, William Dias Belangero

**Affiliations:** 1 Universidade de Campinas (UNICAMP), Faculdade de Ciencias Medicas, Hospital de Clinicas da Unicamp, Campinas, SP, Brazil.; 2 Universidade do Estado do Para (UEPA), Belem, PA, Brazil.; 3 Hospital Osvaldo Cruz, Sao Paulo, SP, Brazil.

**Keywords:** Arthroscopy, Academic Training, Junior Physician, Virtual Reality, Artroscopia, Capacitação Acadêmica, Médico Residente, Realidade Virtual

## Abstract

There is a reduction in the workload of medical residency in orthopedics and a perception that residents can improve their surgical skills without the need to expose patients to risks. Arthroscopies are among the most frequent surgeries in orthopedics and can be trained in a Virtual Reality environment. Our objective is to evaluate the ability of Virtual Reality to develop arthroscopic skills in medical students and orthopedic residents. We conducted the search on Pubmed, Scopus, Embase, and Web of Science platforms. Excluding duplicates, 521 articles remained. We use the Rayyan application to exclude articles outside the scope of the study, and three randomized articles that evaluated the arthroscopic performance of medical students or orthopedic residents with and without training in virtual reality were selected. Regarding time, a tendency towards a decrease in arthroscopic execution time was observed by individuals who trained in VR simulators. Regarding surgical skill, a significant improvement in arthroscopic execution was observed by individuals who trained in VR simulators. Virtual Reality simulators proved effective in developing arthroscopic surgical skills and were an effective alternative in training students and residents. ***Level of Evidence I; Systematic Review and Metanalyses***.

## RESUMO

Há uma diminuição da carga horária de residência médica em ortopedia e uma percepção que os residentes possam aprimorar suas habilidades cirúrgicas sem necessidade de expor os pacientes a riscos. As artroscopias estão entre as cirurgias mais frequentes na ortopedia e podem ser treinadas em ambiente de Realidade Virtual. Avaliar a capacidade da Realidade Virtual para desenvolver habilidade artroscópica em alunos de medicina e residentes. Foram realizadas buscas nas plataformas Pubmed, Scopus, Embase, e Web of Science. Excluindo as duplicatas restaram 521 artigos. Foi utilizado o aplicativo Rayyan para exclusão dos artigos fora do escopo do estudo e selecionados 3 artigos randomizados que avaliavam o desempenho artroscópico de alunos de medicina ou residentes em ortopedia com e sem treinamento em realidade virtual. Em relação ao tempo, foi observado uma tendência de diminuição no tempo de execução artroscópica por indivíduos que treinaram em simuladores de Realidade Virtual. Em relação a habilidade cirúrgica, foi observado melhora significativa na execução artroscópica por indivíduos que treinaram em simuladores de Realidade Virtual. Os simuladores em Realidade Virtual se mostraram eficazes no desenvolvimento de habilidades cirúrgicas artroscópicas e foram uma alternativa eficaz no treinamento de alunos e residentes. ***Nível de Evidência I; Revisão Sistemática e Metanálise***.

## INTRODUCTION

Previous generations of orthopedic surgeons have learned arthroscopy through the model of the apprentice with their preceptor. In this model, as the resident accompanies an experienced surgeon, he gains knowledge and skills, gradually gains more responsibility during surgeries.^1^

Currently there is a tendency to decrease the hourly load of medical residency in the world, which privileges the well-being of the resident, avoiding abuses by some educational centers. On the other hand, there is a decrease in the time available to enhance your learning in some areas. What makes it necessary is a teaching instrument capable of increasing the effectiveness of learning without losing in quality.

Medical competence can be divided into three basic areas: knowledge, skill and attitudes. The knowledge can be developed at any time through theoretical study and benefits from the protected time for this purpose. On the other hand, the development of surgical skills may become impaired as this training requires a learning process that, according to Fits and Posner,^2^ goes through phases of observation, integration and automation. (Table 1)

**Table 1:** Fits-Posner's theory of motor skills acquisition in three stages.

Stage	Objective	Activity	Performance
Cognitive	Understand the goal	Explain and demonstrate	Erratic and distinct steps
Integration	Understand and mechanical performance	Deliberate practice and feedback	More fluid and less interruptions
Automation	Performance with speed, efficiency and precision	Automated performance and requires a minimum of cognition, focusing on refinement	Continuous, fluid and adaptive

Adapted from Reznick.^2^

This learning process evolves from the theoretical knowledge of how a surgery should be performed yet without the ability or motor experience of the procedure, going through motor development following cognition to automation. Here the performance follows agile, effective and accurate. This development occurs through a process of repetition and improvement, which at the beginning of learning, increases the surgical time. This increase in surgical time directly competes with the hospital's goal of optimizing the use of the surgical center and reducing costs.^2^

The learning curve is long and there is some difficulty teaching these skills to residents. The cost of teaching residents to perform arthroscopy is high ($48,000) in the 4 years of residence. Body training is also costly and limited due to degradation of parts and anatomical variations.^1^ It is believed that the learning curve of an "expert" for performing knee arthroscopy is about 170 surgeries performed.^3^

Another concern, even more important, is that residents can improve their surgical skills without exposing patients to risks. They can be trained in a Virtual Reality (VR) environment, with the development of the skill, receiving feedback and improving the skills without exposing the patient to risks.^4,5^ In addition to the skill reducing the procedure time and efficiency of movements, the VR also allows to develop the tactile perception of the learner and can analyze the iatrogenic/chondral lesions "caused" by optics and instruments during training.^5^

Although there is still a long way to go, the field of VR in arthroscopy training has expanded rapidly despite the limited scientific evidence.^6^ In addition, it could become an opportunity for effective training of the resident and without exposing the patient to risk.

This study sought the scientific basis to confirm that VR is effective in teaching and should be increasingly incorporated into residency programs.

## MATERIALS AND METHODS

This systematic review and meta-analysis was carried out according to the PICOS protocol (population: medical students and residents, intervention: VR training, comparison: conventional training, result: score scores and total time, and study design: clinical trials).^7^

### Eligibility criterion

To answer the research question, we need intervention studies (1) (2) in medical students or orthodontists (3) that compare the effect of knee arthroscopy training in VR and non-intervention (controls), reporting at least one of the following results – execution time and score (Global Rating Score-GRS and Fundamentals of Arthroscopic Surgery Training (FAST).

### Search strategy

The systematic search was conducted on the platforms Pubmed (Medline), Scopus, Embase, and Web of Science. The search was carried out in August 2023 with the following combination: (("arthroscopy"[tiab] OR "Arthroscopic Surgery"[tiab] OR "Orthopaedic Surgery"[tiab])) AND (("simulation training"[tiab] OR "virtual reality"[tiab] OR "simulation training"[tiab] OR "Surgical Simulation"[tiab] OR "Residency Triangulation"[tiab] OR "VR simulation"[tiab])).

### Selection of studies

**Figure 1:**
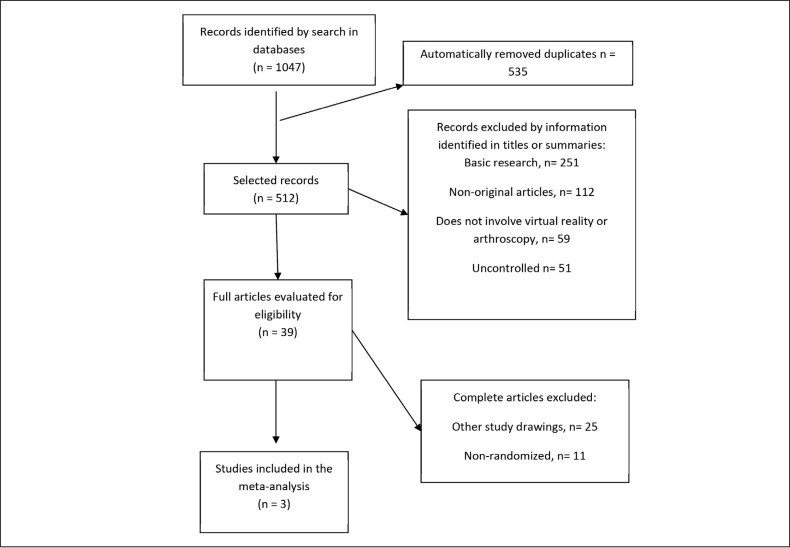
Fluxogram of selection of articles.

209 articles were selected in Pubmed, 367 articles in Scopus, 181 articles in Embase and 290 articles in the Web of Science. All results were exported in RIS format. The duplicates were removed by Software EndNote and 521 articles remained. These were exported to the Rayyan web application and two independent researchers (one resident of the second year of orthopedics and one doctoral student) sorted the papers by title and summary. The conflicts were resolved by a third researcher (MD/PhD). (Figure 1)

The mean, standard deviation and sample size were collected from the intervention and pre- and post-intervention control group for arthroscopic time analysis and arthroscopic ability score.

Statistical analysis

The meta-analysis was performed using the Software Comprehensive Meta-Analysis (Version 3.3.070; Biostat Inc). Two goals-analyses were performed: one for the time of the arthroscopy and another for the score of arthroscopic ability.

## RESULTS

### Studies Selection

1047 articles were selected, excluding 535 duplicates. Of the remaining 512 articles, were excluded through title analysis and summary: 251 for being of basic research; 112 not original; 59 did not involve VR or arthroscopy; 51 were not controlled. 39 remaining full articles to evaluate eligibility. Of these, 25 presented other study drawings and 11 were not randomized. 3 articles were used in the meta-analysis.^8–10^

### Population Characteristics

These meta-analyses totaled 128 volunteers, including 83 medical students and 45 orthodontics residents. The studies compared knee arthroscopy training in VR with control groups that did not carry out VR training. The data were extracted by two researchers (one resident of the second year of orthopedics and one doctoral student) and there were no divergences. The training time was from one hour to eight hours and the performance evaluation was conducted between two weeks and 128 days (Table 2). The quality of the studies was evaluated by the PEDro scale by two researchers (one resident of the second year of orthopedics and one doctoral student) and the divergences were resolved by a third researcher (MD/PhD). (Table 3)

**Table 2:** Characteristics of the studies included.

First author, year	n	Age (y)	Evaluation time	Inclusion criterion	Exclusion criterion
Camp, 20169	Control = 19 Body training = 19 Simulator training = 19	Group 1 = NP Group 2 = NP Group 3 = NP	Pre and post intervention	The 59 residents of the institution were invited.	Residents were excluded if they had no interest or conditions to participate. If they had participated in the study design in some way.
Cychosz, 201810	Control = 21 FAST = 22	Control = 25.8 ± 2.94 FAST = 25.1 ± 1.42	Pre-intervention e1-2 weeks after intervention	Medical school students between April 2016 and April 2017.	Exclusion criteria consist of prior arthroscopic experience, prior training in arthroscopy simulators, inability to use simulators and inability to return after one week for follow-up.
Banaszek, 20178	Desktop Simulator = 16 Virtual Reality = 16 Control = 8	Desktop Simulator = 23.2 Virtual Reality = 25.1 Control = 22.7	Pre-intervention and 5 weeks after the intervention.	NR	NR

Legend: NR: not mentioned in the original article.

**Table 3:** PEDro scale of evaluation of the quality of studies.

First author, year	1	2	3	4	5	6	7	8	9	10	11	Sum
Camp, 20169	No.	Yes	No.	Yes	No.	No.	Yes	No.	Yes	Yes	Yes	6
Cychosz, 201810	No.	Yes	No.	Yes	No.	No.	No.	Yes	Yes	Yes	Yes	6
Banaszek, 20178	No.	Yes	Yes	Yes	No.	No.	Yes	Yes	Yes	Yes	Yes	8

Legend: 1: Specified eligibility criteria; 2: Randomized allocation; 3: Hidden allocation; 4: Similar groups; 5: Blind subjects; 6: Blind Interventor; 7: Blind evaluator; 8: Sample loss less than 15%; 9: Analysis by intent to treat; 10: Statistics comparing groups; 11: Point measurements and variability data.

### Synthesis of Evidence

**Figure 2:**
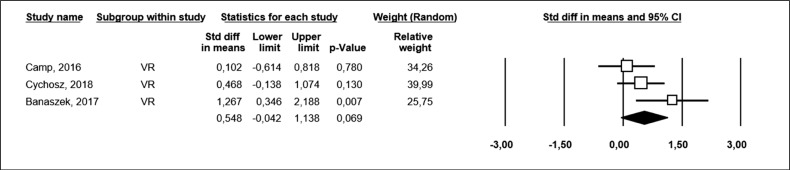
Meta-analysis with the time difference between the VR-trained groups and the control group.

**Figure 3:**
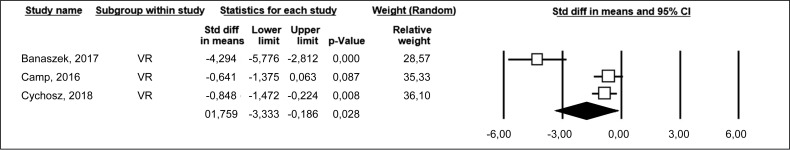
Meta-analysis showing the difference in overall arthroscopic skill scores between the VR-trained group and the control group.

RV training showed a tendency to decrease surgical time. (Figure 2) RV training improved the arthroscopic skills in learners. (Figure 3)

## DISCUSSION

These meta-analyses demonstrated that the training of residents and medical students in VR arthroscopy improved the surgical skills of these learners (Figure 3) and showed a tendency to decrease surgical time. (Figure 2)

In these works, although the time available for training was only one to eight hours, much less than the recommended for arthroscopic training that would be 42 hours/year for three years,^11^ there was significant result in surgical ability. A longer training time may be related to an enhancement of learning by the Fits-Posner theory.^2^ Jacobsen et al demonstrated that a greater number of hours of training associated with performing more elaborate activities in arthroscopy was more effective in psychomotor development.^12^ Beaudoin et al demonstrated that longer training time is related to better arthroscopic performance.^13^

Another important point is the training of a skill that is not being recruited/practiced. Among the articles included in these meta-analyses, Camp et al.^9^ showed 128 days between four-hour training and re-evaluation. This time without training can be one of the causes of the non-expressive result of VR training. Continued training of surgical skills is indicated to avoid this psychomotor deterioration.^14^ The advantages related to VR are the non-exposure of patients to risks during training, development of psychomotor skills including tactile and savings in relation to training in real patients and corpses,^1,2^ The skills developed in VR had their transfer to real arthroscopy in surgical center validated, demonstrating final benefit to the patient.^15^ RV has other applications besides surgical ability, it can be performed the training of the fixation of the eye during arthroscopy^16^ or even review a surgery with VR demonstrating the necessary care and reviewing the step-by-step surgery.^17^

Other desk training models (physical parts simulating a knee or a cavity) are cheaper, ^18–20^ allow the development of psychomotor skills, but were not part of the scope of this study. Thinking of the reality of the country, these systems could be useful in training residents and an economically more viable alternative. This method can improve the teaching of arthroscopy and is suitable for use in most medical residency services in orthopedics.

There are several limitations to this study. First, the lack of randomized studies. The methods of evaluation of surgical skills are different from each other, which makes it impossible to use a larger number of articles. Another important point is that there are several different VR simulators for training, but each has its own characteristics that can lead to an intervention or evaluation bias.

## Conclusion

The training of medical students and orthodontics residents through VR has been shown to be effective in improving the arthroscopic surgical skills of the knee.
